# The Pitfalls of Abnormal Laboratory Value Interpretation in Vaccine Clinical Trials: The Example of Asymptomatic Transient Neutropenia

**DOI:** 10.1007/s40268-021-00370-3

**Published:** 2021-12-18

**Authors:** Venanzio Vella, Johannes E. Schmidt, Giulia Luna Cilio, Iris De Ryck, Audino Podda, Valentino Conti, Joachim Auerbach

**Affiliations:** 1 GSK Vaccines R&D Center, Via Fiorentina 1, 53100 Siena, Italy; 2 GSK Vaccines Clinical Safety and Pharmacovigilance (VCSP) Department, Siena, Italy; 3 GSK Vaccines Institute for Global Health S.r.l. (GVGH), Siena, Italy

## Abstract

Hematological and clinical chemistry measurements are an integral part of vaccine safety monitoring. While adopting a conservative approach is important to exclude potential risks for patients, the rationale and methodology underlying the assessment of given adverse events have to be well grounded to avoid raising unfounded concerns. Using asymptomatic transient neutropenia as an example, this paper aims to address the complexity of interpreting abnormal hematological values in vaccine clinical trials and to evaluate the validity of using neutrophil count cut-off points to assess neutropenia in the context of safety monitoring. The validity of the neutrophil count cut-off point methodology was assessed in terms of content validity (i.e., the extent to which a single neutrophil count below the cut-off point corresponds to a clinically significant adverse event), criterion validity (i.e., the extent to which a neutrophil count below a given cut-off point correlates with another manifestation of neutropenia, namely bacteremia), and construct validity (i.e., the exactness of the assumption that a neutrophil count below a given cut-off point corresponds to a reactogenic event caused by the vaccination). We argue that, because of within-individual physiological fluctuations, variations according to population demographics, and poor predictive potential with regard to neutropenia-associated infection, the application of the cut-off point methodology to neutropenia safety monitoring presents major limitations. Based on this assessment, we conclude that hematological laboratory values must be evaluated on a case-by-case basis by investigators to determine their clinical significance.

## Key Points

**Table:** 

Cut-off points are commonly used to determine if laboratory values, such as the number of white blood cells like neutrophils in blood samples, are abnormal and represent a safety concern during clinical trials.
By using a harmless condition called asymptomatic transient neutropenia as an example, we show that the current use of cut-off values is not adequate to highlight abnormal drops in neutrophil counts.

## Introduction

Neutrophils are the body’s front-line defense against pathogens [1, 2]. Originating from bone marrow stem cells [3], neutrophils are the most abundant type of white blood cells [1] and constitute the initial cellular component of the inflammatory response, through cytokine release and phagocytosis activities [1]. Because of their short lifespan, i.e., from a few hours to a few days [4, 5], neutrophil counts display high variability between individuals and fluctuate daily in a given individual [6]. Despite these variations, drops in neutrophil count below the lower normal limit, a phenomenon termed neutropenia, can result in increased risks of infection [6]. However, decreases in neutrophil count also occur as a result of asymptomatic transient neutropenia (ATN), a temporary neutrophil count below a defined cut-off point in asymptomatic healthy individuals that returns to the “normal” range of values upon further testing [7], or pseudoneutropenia, the redistribution or agglutination of neutrophils [6].

Abnormal hematological findings are monitored in healthy participants at specific timepoints during phase I clinical trials to evaluate whether investigational medicinal products (IMPs), such as candidate vaccines, could result in health hazards [7]. For this safety monitoring process to be meaningful, it is crucial to define appropriate reference values for the evaluation of specific adverse events (AEs) [8].

Several guidelines, defined based on cut-off points in neutrophils/mm^3^, are used for the classification of neutropenia in vaccine clinical trials (Table 1). While these cut-off points are likely to highlight low neutrophil counts that warrant further investigation, they present several weaknesses. The US Food and Drug Administration Guidance for the Industry [9] was developed for trials in adult and adolescent participants, and is not appropriate for studies carried out in children, who naturally present with lower neutrophil counts [6]. In addition, the previously mentioned classifications fail to account for variations observed across ethnicity, sex, and age [10]. Furthermore, there may be a lack of a substantive rationale underlying the use of these cut-off points with regard to neutropenia, notably because of the unclear association between neutropenia and increased risk of infection and the fact that the neutropenia observed in vaccine clinical trials is generally transient and not of clinical significance [7, 11].

**Table 1:** Guidelines for the classification of neutropenia

Neutropenia status	US FDA guidance for the industry [9]	BMJ best practice assessment [6]	WHO [19]
Neutropenia-negative values (neutrophils/mm^3^)	> 2000	> 1500	≥ 1800
Neutropenia-positive values (neutrophils/mm^3^)	1500–2000 (grade 1; mild)	1000–1500 (mild)	< 1800
	1000–1499 (grade 2; moderate)	500–999 (moderate)	
	500–999 (grade 3; severe)	200–499 (severe)	
	< 500 (grade 4; potentially life threatening)	< 200 (very severe)	

*US FDA* United States Food and Drug Administration, *WHO* World Health Organization

Although a conservative approach to safety monitoring is important, the rationale and methodology underlying the assessment of given AEs have to be robust and based on solid evidence to avoid raising unfounded concerns. The ‘cut-off point’ approach is likely to highlight dangerously low levels of neutrophil counts and potentially life-threatening cases of neutropenia. However, it fails to factor in several important aspects of the condition, which, in turn, may result in flagging of non-clinically significant events, such as ATN or pseudoneutropenia.

**Fig. 1:**
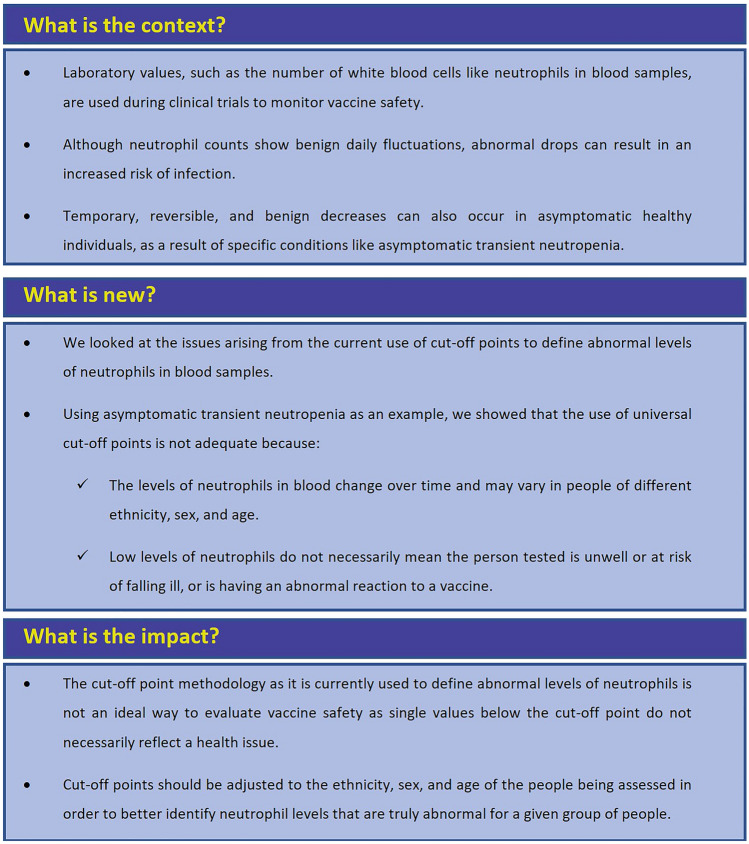
Plain language summary

Using ATN as an example, this paper addresses the complexity of interpreting abnormal hematological values in vaccine clinical trials using the cut-off point methodology. A summary contextualizing the outcomes of this publication is displayed in the Plain Language Summary (Fig. 1).

## Assessing ATN in Vaccine Trials According to Validity Criteria

Validity is defined as the degree to which a test measures what it purports to measure [12]. In the context of safety monitoring during vaccine clinical trials, the cut-off point methodology aims to measure the clinical significance of a highlighted AE, and its validity for the classification of neutropenia can be assessed in terms of:

- Content validity [12], i.e., to what extent does a single neutrophil count below the cut-off point correspond to a clinically significant AE?
- Criterion validity [12], i.e., to what extent does a neutrophil count below a given cut-off point correlate with infection, evaluated using bacteremia as a proxy (which is an external manifestation of the phenomenon being assessed, namely neutropenia)?
- Construct validity [12], i.e., how exact is the assumption that a neutrophil count below a given cut-off point corresponds to a reactogenic event?

Reassessing the application of the cut-off point methodology to ATN with regard to these validity criteria is critical to clarify whether isolated laboratory neutrophil counts below a given cut-off point in asymptomatic subjects correspond to abnormal values or AEs.

### Content Validity

An AE is defined as any untoward medical occurrence associated with the use of an IMP, whether it is drug related or not [13]. In the context of the assessment of neutropenia as an AE using the cut-off point methodology, it is important to determine if a single neutrophil count below the cut-off point corresponds to a clinically significant AE. The threats to the content validity of using the cut-off point methodology in the context of ATN (pseudoneutropenia, variability associated with neutrophil counts, and the expected prevalence of ATN for a specific population) are detailed in the sections below.

#### Pseudoneutropenia

Pseudoneutropenia, defined as a single value below the cut-off point due to the redistribution or agglutination of neutrophils, can occur as a result of (1) a shift of neutrophils to a marginated pool (the adherence of neutrophils to capillary and venule endothelium), leading to a decrease in neutrophils circulating in blood (circulating pool), (2) the agglutination of neutrophils within the circulating pool, leading to a reduced neutrophil blood count; or (3) the agglutination of neutrophils after blood sampling due to the use of ethylenediaminetetraacetic acid to prevent blood coagulation [6]. ATN and pseudoneutropenia are virtually indistinguishable based on neutrophil count values, and a diagnosis of neutropenia cannot be established with confidence until it has been confirmed by repeat testing and assessment by a medical professional [6].

#### Physiological Fluctuations in Neutrophil Count

The aim of safety monitoring is to detect signs of physiological organ system dysfunction or abnormality that might indicate a reaction to the vaccine and thus represent a potential safety concern. To avoid interpreting physiologically normal events as reactogenic, it is critical that the measurement used to highlight these abnormalities is reliable, i.e., stable when the measurement is repeated under identical conditions [12, 14]. In the context of neutropenia, a single neutrophil count below a defined cut-off point should ideally be a reliable indication of neutropenia. However, as neutrophil count is notoriously variable, this does not appear to be the case.

High physiological within-participant variation of neutrophil counts was reported based on laboratory test data from the 1999–2002 National Health and Nutrition Examination Survey (NHANES) in the civilian noninstitutionalized US population [15]. In this study, neutrophil counts in two blood samples from the same subject displayed high variation compared with 17 other hematological parameters tested, and were as likely to increase or decrease compared to the baseline value [15]. However, in the context of safety monitoring, declines are generally the object of more attention than increases, as they can be associated with an increased risk of infection. As a result, a physiologically normal, transient decrease in neutrophil count following vaccine administration might be erroneously flagged as an AE linked to the IMP.

#### Expected Prevalence of ATN for a Specific Cohort of a Given Size

The content validity of the neutropenia safety assessment methodology is also dependent on the definition of unexpected/abnormal neutrophil count values. Abnormal values can be defined on the basis of either a low probability of such values occurring in a given population (e.g., values below or above 2 standard deviations from the mean) [16] or on the increased probability that a given disease or condition is present if such values are reached [8]. It is therefore critical to assess the cut-off points used to define abnormal neutrophil counts in the context of clinical trials with regard to these criteria.

Like any other laboratory value, neutrophil counts follow a population-specific probability distribution. The 1999–2004 NHANES found that the prevalence of a non-clinically significant reduction in neutrophils < 1500/mm^3^ in the US population varied according to age, sex, and ethnicity [10]. The prevalence of neutrophils < 1500/mm^3^ was 1.2% overall, with a variable prevalence in different ethnic groups: 6.7% for African American male individuals, 3.6% for African American female individuals, 0.9% for White male individuals, and 0.6% for White female individuals. The prevalence of neutrophils < 1000/mm^3^ was 0.6% for African American participants and 0.1% for White participants, and as high as 3.6% in the 1–2 years age group and 1.7% in the 3–5 years age group of African American participants. Consequently, depending on cohort size and characteristics, values as low as < 1000/mm^3^ are expected to occur according to the normal distribution of neutrophil counts in healthy participants, without these being signs of reactogenic events or pathologic conditions.

**Fig. 2:**
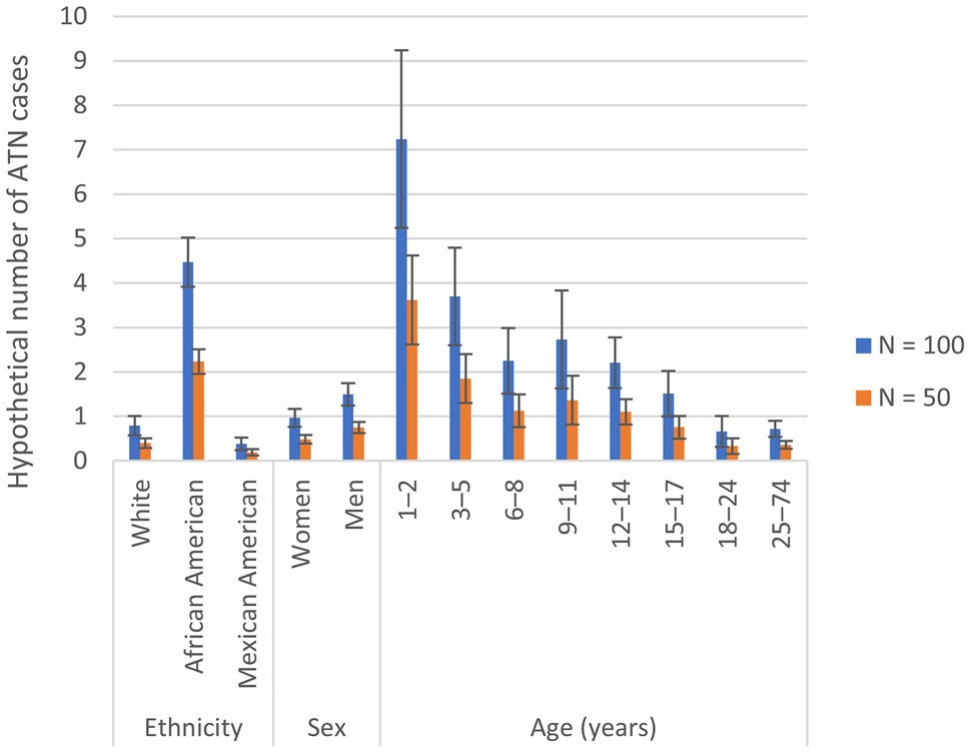
Expected number of asymptomatic transient neutropenia (ATN) events, as defined by using a < 1500 neutrophils/mm^3^ cut-off point, in cohorts of either 50 healthy participants (*N* = 50) or 100 healthy participants (*N* = 100) according to ethnic group, sex, and age. *N* number of participants in the cohort. *Error bars* represent 95% confidence intervals. Hypothetical numbers of subjects with neutrophils < 1500/mm^3^ per 50 or 100 healthy participants were calculated by multiplying the prevalence of neutrophils < 1500/mm^3^ for White (0.79%), African American (4.47%), and Mexican American (0.38%) participants, women (0.97%), men (1.50%), participants of age groups 1–2 (7.24%), 3–5 (3.70%), 6–8 (2.25%), 9–11 (2.73%), 12–24 (2.21%), 15–17 (1.51%), 18–24 (0.66%), and 25–74 years (0.72%) estimated in the National Health and Nutrition Examination Survey (NHANES) [10] study by 50 (for a population of 50 healthy participants) or 100 (for a population of 100 healthy participants)

The occurrence of individual values under a standard cut-off point is therefore meaningless unless cohort size and demographic characteristics have been factored in when defining the cut-off point. To further illustrate this issue, we have applied the expected prevalence of neutrophils < 1500/mm^3^ from the NHANES [10] to theoretical cohorts of 100 healthy participants of a given ethnicity, sex, and age, recruited in a hypothetical trial (Fig. 2, blue bars). Depending on ethnicity and age, the number of abnormal values per 100 healthy subjects would range from < 1 in a cohort consisting of White participants, to > 4 in a cohort consisting of African American participants and > 7 in a cohort of children aged 1–2 years.

Further biases to the cut-off point approach may occur in clinical trials assessing vaccinated and placebo groups of unequal cohort sizes (Fig. 2, blue vs orange bars). Given that the expected number of healthy participants in a group displaying neutrophil counts below the cut-off point increases with the group size, it is expected that a group comprising 100 participants would report around twice as many cases below the cut-off point compared with a cohort of 50 participants, independently of any causal association with the administered vaccine.

Even more striking population-specific biases can occur if the populations being compared are unbalanced both with regard to demographic characteristics and sizes. For example, a population of 50 healthy White adults will display very few participants with neutrophil counts < 1500/mm^3^ compared with a population of 100 healthy African American infants, where a higher number of potential neutropenic events would be flagged during clinical trial safety monitoring, even though these are to be expected based on population characteristics. Population-specific probabilities therefore inform investigators about the likelihood that a low neutrophil count would reflect an unexpected or an expected event.

### Criterion Validity

Neutrophils are critical for the prevention of infections [6], and it is a logical assumption that a reduction in neutrophil count, even transient, could increase the risk of infection. The criterion validity of the neutropenia cut-off point methodology can be assessed by comparing the predictive power of the neutrophil count cut-off points against the gold standard for evaluating infection, i.e., bacteremia.

Correlations between several hematological parameters, including neutrophil count and bacteria-positive blood cultures were evaluated by De Jager et al. in a retrospective study of the health records of adult patients admitted to the emergency department [11]. Patients with bacteria-positive and bacteria-negative blood cultures were age and sex matched, while patients with hematological disease and patients receiving chemotherapy or glucocorticoids were excluded to avoid possible confounding factors. The analyses showed no statistically significant differences in mean neutrophil counts in patients with bacteria-positive or bacteria-negative blood cultures, suggesting that this hematological measurement is a poor predictor of bacterial infection. This low predictive power was further supported by the receiver operating characteristic curve for neutrophil counts (which evaluates the sensitivity and specificity of neutrophil counts in discriminating between cases of bacteremia and non-bacteremia) and the area under the curve, which, at 0.57 for the neutrophil count, was only a fraction above the threshold for random class attribution (area under the curve of 0.5) [17]. Therefore, although neutropenia has been reported as an AE in over 30 phase I and II vaccine trials [7], there is a lack of evidence that transient neutropenia is indeed a reactogenic sign and/or is associated with a higher risk of infection for the affected individuals.

### Construct Validity

The construct validity of the neutrophil count cut-off point methodology requires the following assumptions to be true:

- A decline in neutrophil count below a cut-off point is potentially a sign of a reactogenic event.
- A decline in neutrophil count below a cut-off point is potentially associated with a higher risk of another manifestation of the condition being assessed, e.g., infection.

In addition, for a given IMP and an existing pathological condition, a strong biological rationale underlying these assumptions would strengthen the construct validity of a cut-off point methodology.

The cut-off methodology shows high construct validity when applied to the monitoring of neutropenia associated with the myelotoxic effects of chemotherapeutic agents [18]. In this case, a clear biological rationale for this methodology exists in the cause–effect relationship between the myelotoxic drug and decreases in neutrophil counts [18]. The construct validity of this approach is further increased by the relatively stringent cut-off point used, i.e., 500–1000 neutrophils/mm^3^ for mild neutropenia and < 500 neutrophils/mm^3^ for severe neutropenia (the likelihood of a neutrophil count below this cut-off point being a random event is low, and may therefore indicate toxicity) [18]. In addition, construct validity benefits from the strong association of low neutrophil counts with fever (febrile neutropenia), which occurs in up to 80% of patients with hematologic malignancies treated with chemotherapy, and are highly predictive of a drug safety issue [18].

However, there are considerable differences between using neutropenia for monitoring myelotoxicity in patients with cancer and using it in healthy individuals in vaccine trials. While the use of the neutrophil count cut-off methodology shows strong construct validity when assessing AEs resulting from chemotherapy (e.g., febrile neutropenia), the same conclusion cannot be drawn for healthy individuals participating in vaccine clinical trials. Indeed, as discussed above, a decline in neutrophil count below the cut-off point is not necessarily linked to a reactogenic event (see Sect. 2.1), nor is it strictly associated with a higher risk of infection (see Sect. 2.2).

## Discussion and Conclusions

In the context of safety monitoring during vaccine clinical trials, hematological data are critical to highlight potential safety concerns associated with an IMP. This should occur as early as possible during vaccine clinical development [7], to minimize the risks to the trial participants and to allow an accurate characterization of the product’s benefit-risk profile.

While neutropenia can be a life-threatening condition warranting further follow-up, the arbitrary cut-off point methodology used to detect this AE does not seem adequate and applicable in all cases. Using arbitrary cut-off points to categorize neutropenia severity is not inherently wrong; however, its validity relies on the metric being synonymous with an increased risk. This is not the case for ATN, as the cut-off point methodology does not enable a strict discrimination between ATN and pseudoneutropenia, nor does it account for intra-individual and inter-individual neutrophil count variability. Moreover, there is a startling lack of published data supporting the link between neutrophil counts under given cut-off points and an increased risk of infection or other physiopathological conditions. Although neutropenia is not an uncommon occurrence following vaccination, post-vaccination neutropenia is generally transient and not of clinical significance [7]. While neutropenia can be seen as a reactogenic event following vaccination in such cases, similar events are also observed in placebo groups and might be the result of neutrophils being temporarily drawn to the injection site (pseudoneutropenia) rather than a reaction to the IMP [6]. The lack of robust validity or clinical rationale supporting the current methodology to assess neutropenia as an AE raises the question of why it is still used.

Although conclusions drawn in the present opinion piece are built on first-hand experience with neutropenia and ATN cases, the pitfalls and reasoning described here can and should be applied to any laboratory cut-off point methodology used in the context of clinical trial safety monitoring. Reviewing approaches to IMP safety monitoring and applying corrective strategies wherever relevant appears all the more important in the context of the global COVID-19 pandemic. Indeed, COVID-19 vaccines are being developed and deployed at much faster rates than was ever the case for other vaccines in the past, with safety monitoring of these drugs being carried out simultaneously with an extremely high degree of public scrutiny regarding potential AEs. It is therefore paramount that methodologies employed for safety monitoring of these vaccines strike the right balance between not raising unfounded safety concerns that might stoke hesitancy and flagging actual vaccine-associated safety issues.

The safety of clinical trial participants takes precedence over any other aspect of a clinical trial; however, the monitoring of hematological values may need to be re-evaluated in light of current knowledge of the conditions and/or disorders they are meant to serve as proxy for, in order for safety conclusions to be meaningful. Although severity grading contributes positively to the standardized reporting of AEs, it should address the following questions to assess its relevance with regard to the evaluation of IMP-related safety issues (e.g., neutropenia) during clinical trials:

- Are the normal ranges of hematological values used as proxy for given conditions/disorders (e.g., neutrophil counts in the case of neutropenia assessment) defined appropriately given the age, sex, and ethnicity of the participants under study?
- Is the occurrence of an out-of-range hematological value (e.g., low neutrophil count in the case of neutropenia assessment) unexpected for a population of given characteristics and size?
- Is an occurrence of an out-of-range hematological value (e.g., low neutrophil count in the case of neutropenia assessment) clinically significant according to the investigator’s clinical judgment?
- What actions should be taken if the AE associated with a given hematological value (e.g., neutropenia as defined by neutrophil count) is not deemed to be the sign of a safety issue?

Answering the above questions will provide a basis for critically assessing out-of-range hematological values according to the context they were obtained in, thereby accounting for sources of variation (such as population characteristics and size, as well as intra-individual and inter-individual variability) and reducing the risk of overinterpretation of results (i.e., raising unfounded safety concerns). There is currently no evidence indicating that an isolated neutropenic value under a given cut-off point in healthy participants is predictive of a clinically relevant increased risk for patient safety. However, as ATN may still represent a reactogenic event following vaccination, it is important for clinicians to have a clear framework to assess hematological laboratory values. In turn, this entails that asymptomatic and clinically significant forms of the conditions/disorders being assessed (e.g., ATN vs neutropenia) have to be as clearly defined and readily distinguishable as possible. In the case of neutropenia, it is our opinion that clinically significant forms of the condition should meet the following requirements: (1) that neutrophil counts should only be considered as out of range if they are below a cut-off point adequately defined (based on a thorough investigation and analysis, while being mindful of the limitations and validity criteria described in our article) for the individual or population being assessed, (2) that an out-of-range neutrophil count does not return to the adequately defined range upon repeat testing, and (3) that no other clinical manifestations of neutropenia (e.g., infection or bacteremia) be observed concomitantly with an out-of-range neutrophil count value. Furthermore, it is also important that investigators consider out-of-range hematological values in the context of the clinical trial design. For instance, a statistically significant difference in the number of out-of-range values for a given hematological parameter in the treated group compared with the placebo will warrant further scrutiny.

As mentioned above, a similar rationale to what we present and discuss here applies to other conditions and/or disorders evaluated using hematological laboratory values. It is our hope that the discussion presented here may promote the critical re-evaluation of how specific laboratory-assessed parameters (such as those used to diagnose AEs [i.e., thrombocytopenia and coagulation dysfunctions] reported in COVID-19 vaccine trials) should be interpreted. Hematological laboratory values such as neutrophil count and cut-off points should not be used alone to highlight specific issues during clinical trial safety monitoring, but in association with the investigator’s assessment of their clinical significance, while simultaneously considering the limitations described in the present manuscript.
